# A novel bio-electro-Fenton system with dual application for the catalytic degradation of tetracycline antibiotic in wastewater and bioelectricity generation

**DOI:** 10.1039/d1ra04584a

**Published:** 2021-08-09

**Authors:** Fatemeh Soltani, Nahid Navidjouy, Hassan Khorsandi, Mostafa Rahimnejad, Saber Alizadeh

**Affiliations:** Department of Environmental Health Engineering, School of Public Health, Urmia University of Medical Sciences Urmia Iran n.navidjouy@gmail.com n.navidjouy@umsu.ac.ir +98 9143489617; Biofuel and Renewable Energy Research Center, Department of Chemical Engineering, Babol Noshirvani University of Technology Babol Iran; Faculty of Chemistry, Bu-Ali-Sina University Hamedan Iran

## Abstract

In this new insight, the potential application of the eco-friendly Bio-Electro-Fenton (BEF) system was surveyed with the aim of simultaneous degradation of tetracycline and *in situ* generation of renewable bioenergy without the need for an external electricity source. To shed light on this issue, catalytic degradation of tetracycline was directly accrued *via in situ* generated hydroxyl free radicals from Fenton's reaction in the cathode chamber. Simultaneously, the *in situ* electricity generation as renewable bioenergy was carried out through microbial activities. The effects of operating parameters, such as electrical circuit conditions (in the absence and presence of external resistor load), substrate concentration (1000, 2000, 5000, and 10 000 mg L^−1^), catholyte pH (3, 5, and 7), and FeSO_4_ concentration (2, 5, and 10 mg L^−1^) were investigated in detail. The obtained results indicated that the tetracycline degradation was up to 99.04 ± 0.91% after 24 h under the optimal conditions (short-circuit, pH 3, FeSO_4_ concentration of 5 mg L^−1^, and substrate concentration of 2000 mg L^−1^). Also, the maximum removal efficiency of anodic COD (85.71 ± 1.81%) was achieved by increasing the substrate concentration up to 2000 mg L^−1^. However, the removal efficiencies decreased to 78.29 ± 2.68% with increasing substrate concentration up to 10 000 mg L^−1^. Meanwhile, the obtained maximum voltage, current density, and power density were 322 mV, 1195 mA m^−2^, and 141.60 mW m^−2^, respectively, at the substrate concentration of 10 000 mg L^−1^. Present results suggested that the BEF system could be employed as an energy-saving and promising technology for antibiotic-containing wastewater treatment and simultaneous sustainable bioelectricity generation.

## Abstract 2

In this new insight, the potential application of the Bio-Electro-Fenton system was surveyed with the aim of simultaneous degradation of tetracycline and *in situ* generation of renewable bioenergy without the need for an external electricity source.

## Graphical abstract

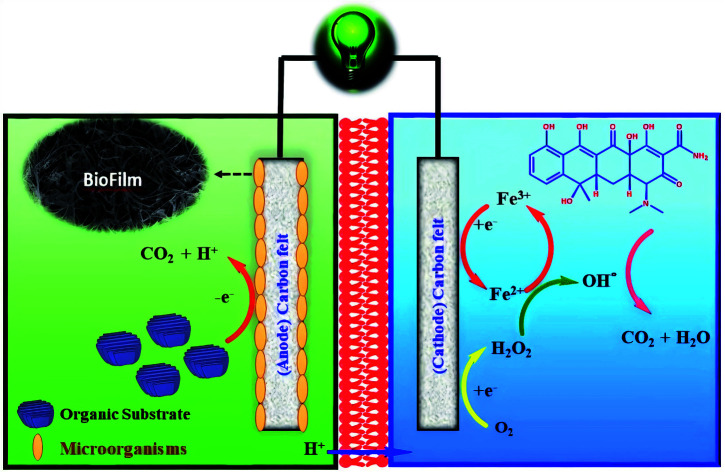

## 1. Introduction

Recently, environmental pollution and energy demand have been the two important issues globally owing to the rapid rise in population growth and industrialization.^1,2^ Energy crisis has risen due to the limited access to fossil fuels and their environmental impacts.^3^ Hence, clean fuels, such as biofuels, fuel cells, hydrogen, and biomass-based energy, as renewable fuels without any pollution, are suitable alternatives.^2,4,5^ Moreover, water pollution is a major global concern due to the problems caused by the entry of recalcitrant organic pollutants in wastewater into to the aqueous environments. Thus, pharmaceutical residues in wastewater are a source of serious threat to the environment.^6–8^ Antibiotics are significant pharmaceutical chemical compounds, which are nowadays identified as emerging active environmental pollutants.^9,10^ The widespread consumption of antibiotics as human and veterinary medicines with the annual use of 100 000 to 200 000 tons and increasing human activities are responsible for the persistent presence of antibiotic pollutants in the natural environment.^9,11,12^ Public concern on serious environmental impacts of antibiotics has raised in recent years.^13^ In fact, the inappropriate disposal of antibiotics continuously into water matrices can not only lead to the potential risk of harm for human health, as well as aquatic or terrestrial ecosystems in the long term but also affects drinking water supplies.^11,14^ Among the common antibiotics, tetracycline (TC) was selected as the target pollutant in this study due to its extensive global usage as human and veterinary medicine against infection, non-biodegradable and toxic nature, and also its occurrence in the environment.^7,15–17^ Typically, the concentrations of TCs have detected at around 100 μg L^−1^ in hospital wastewater, 1 μg L^−1^ in domestic wastewater, and 0.15 μg L^−1^ in ground and surface water.^18,19^ Hence, even low amounts of antibiotic residues in water bodies can pose serious environmental problems.^20^ Therefore, the efficient removal of antibiotics from the water and wastewater sources is necessary.^14,21^

Among the technologies, advanced oxidation processes (AOPs) as potentially powerful technologies have been successfully used for the removal of antibiotics and other refractory pollutants, such as photocatalysis,^22–24^ photo-Fenton,^25,26^ ozonation^21,27^ and electro-Fenton processes.^28,29^ AOPs as oxidation technologies are based on the production of hydroxyl radicals (˙OH), which have a strong electrochemical oxidant potential.^30^ Especially, electro-Fenton process (EF) is one of the most common electrochemical advanced oxidation processes (EAOP) with high efficiency and strong degradation capacity that has attracted considerable attention.^31,32^ Notably, in this process, homogeneous free ˙OH can be generated *via* the fundamental chemical reaction between *in situ* electrogenerated hydrogen peroxide (H_2_O_2_) and ferrous ions (Fe^2+^) to degrade or mineralize a wide variety of organic pollutants into non-toxic or low molecular weight compounds.^12,33,34^ However, applying external energy in EF process can result in high operating costs, particularly if complete mineralization is required.^15^

In recent investigations, the renewable bioenergy technologies, such as bio-electrochemical systems (as microbial fuel cell (MFC) and microbial electrolysis cell (MEC)),^5,35–37^ nanotechnology,^3,38^ photocatalytic methods,^39–41^ biological and biochemical technologies,^42^ and other methods have been considered for bioenergy generation and resource recovery. In addition, at present, eco-friendly solar-driven photocatalytic processes have also been reported for adsorption and degradation of recalcitrant organic pollutants and green energy generation under solar light irradiation without the application of an external energy input.^43–48^ As a consequence, a demand has emerged to research eco-friendly and green technologies for the harmful organic pollutant removal from wastewater sources and clean energy production.^2,49^

Among the various green technologies, MFC systems combined with the cathodic EF process have been recently suggested as bio-electro-Fenton (BEF) systems for efficient removal of refractory organic pollutants and simultaneous bioelectricity generation similar to the mentioned methods.^50–52^ These properties drew most researchers' attention to the BEF systems as a promising alternative and energy-saving approach.^53,54^ In BEF systems, biomass as an organic substrate is oxidized by electrochemically active bacteria through bio-electrochemical reactions in the anaerobic anodic chamber, so no external electricity supply is required as opposed to the EF process.^50,55,56^ Then, the produced bio-electrons and protons from the microbial metabolism in the oxidation of biologically degradable substrate are transferred simultaneously to the cathodic compartment *via* the external circuit and proton exchange membrane, respectively.^52,57,58^ H_2_O_2_ is also formed through the two-electron reduction of electron acceptors (O_2_) (eqn (1)) in the cathodic chamber, which reacts with the iron catalyst to generate ˙OH through eqn (2) and the EF oxidation occurs. Consequently, the oxidative degradation of recalcitrant organic pollutants to oxidation products is carried out by non-selective and highly active ˙OH radicals with strong oxidizing power (eqn (3)).^12,59–61^

1 O_2_ + 2H^+^ + 2e^−^ → H_2_O_2_

2 Fe^2+^ + H_2_O_2_ → Fe^3+^ + ˙OH + OH^−^

3 Recalcitrant organic pollutants + ˙OH → H_2_O + CO_2_

Accordingly, BEF systems can not only produce H_2_O_2_ in the cathode to remove recalcitrant organic pollutants but also recover green energy *via* biodegradation of substrates such as glucose and acetate in the anode.^62,63^ Generally, this bio-electrochemical process of recalcitrant organic compound degradation is considered innovative in the present framework for the treatment of wastewater containing antibiotic pollutants.^64^

So far, the removal of various recalcitrant organic pollutants in the cathodic chamber of the BEF system has been considerably investigated, including *p*-nitrophenol,^65,66^ azo dyes,^67,68^ triphenyltin chloride,^59^ NSAIDs.^69^ Xu *et al.*^53^ employed a BEF system for the degradation of 17α-ethynyl-estradiol and 17β-estradiol and the absorption on the surface of electrode and reactor along with the degradation by ˙OH in the cathode chamber, which resulted in the degradation of 81% of 17β-estradiol and 56% of 17α-ethynyl-estradiol during 10 h and the system achieved a maximum power output of 4.35 W m^−3^. Wang *et al.*^70^ also demonstrated that the BEF process could be an attractive method for arsenite oxidation and current generation. In another study, Zhang *et al.*^57^ investigated the feasibility of paracetamol removal in the MFC–Fenton system, where the degradation efficiency of paracetamol was 70% due to the decomposition by hydroxyl radicals. Ling *et al.*^68^ reported the removal efficiency of methyl orange dye from 73.9% to 86.7% by Fenton's reactions in BEF system equipped with Fe@Fe_2_O_3_/active carbon felt (ACF) composite cathode.

To our knowledge, most bio-electrochemical systems studies have focused on antibiotic removal in the anode chamber, and less attention has been paid to their removal in the cathode.^18,71,72^ Moreover, the results of previous studies have shown that substrate concentration such as glucose and acetate, in addition to impacting the bioelectricity generation, can also affect the degradation of recalcitrant organic pollutants.^73,74^ Hence, this is the first study that separately investigated the effect of the high substrate concentration on tetracycline degradation in the cathode chamber of the BEF system. Therefore, the MFC system was chosen to power the EF process as a BEF system for the degradation of antibiotic pollutants.

In the present study, the feasibility of the double-chamber BEF reactor for the TC degradation as the model pollutant under different operational conditions was demonstrated. To this end, this study examined the degradation efficiency of TC under various electrical circuit conditions. Then, the effect of substrate concentrations on the removal efficiency of anodic COD and cathodic TC, as well as bioelectricity generation, was determined. Finally, the influence of the main cathodic operating parameters, such as pH and FeSO_4_ concentrations, on TC degradation efficiency and the relationship between changes in pH and FeSO_4_ concentrations with electricity generation were investigated. These results are expected to provide an energy-efficient and environmentally friendly approach for the TC degradation in the BEF system.

## 2. Materials and methods

### 2.1. Chemical and materials

TC was purchased from HAKIM Pharmaceutical Co. (Tehran, Iran) and used in the experiments. Different chemicals, such as potassium dihydrogen phosphate (KH_2_PO_4_), dipotassium hydrogen phosphate (K_2_HPO_4_), ammonium chloride (NH_4_Cl), glucose (C_6_H_12_O_6_, H_2_O), calcium chloride (CaCl_2_), magnesium sulfate (MgSO_4_), potassium chloride (KCl), sodium chloride (NaCl), sulfuric acid (H_2_SO_4_ 95–97% purity), hydrochloric acid (HCl 37%), sodium hydroxide (NaOH), Nafion 117 (Sigma-Aldrich), peptone, yeast, heptahydrated ferrous sulfate (FeSO_4_·7H_2_O) catalyst, anhydrous sodium sulfate (Na_2_SO_4_), acetonitrile, and methanol (HPLC grade) were purchased from Merck Company and used without extra purification. All aqueous solutions were made daily with distilled water at room temperature. All the chemicals used in these experiments were analytical grade.

### 2.2. Bio-electro-Fenton reactor configuration and operation

**Fig. 1:**
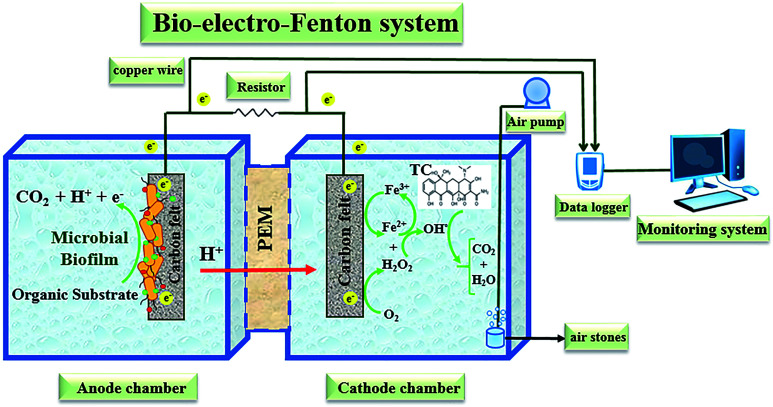
Schematic diagram of the bio-electro-Fenton system reactor.

A dual-chambered bio-electro-Fenton reactor constructed from plexiglass with anodic and cathodic working volumes of 450 mL each was used in this study (Fig. 1). Carbon felt as cathode and anode electrodes with a surface area of 15 cm^2^ for each (50 mm length × 30 mm width × 3 mm thickness) were used and were separated by a cation exchange membrane (Nafion 117, Sigma-Aldrich, USA) to increase H^+^ diffusion.^73^ In order to pretreat the electrodes, they were soaked for 20 min in acetone solution and boiled in 0.1 M HCl for 15 min and eventually washed with water in order to remove the potential foreign contaminants from the carbon felt's surfaces.^75^

A copper wire with 0.5 mm diameter as current collector connected the two electrodes by passing through an external resistor and an analogue digital data logger (Danesh Gostar Hamgam Ba Sanat Company (Babol, Iran)) connected to the computer for recording output voltage.^58^

The mixed active microorganisms of the anaerobic sludge were collected from an anaerobic digester tank of the municipal wastewater treatment plant of Urmia city as inoculum and glucose as the carbon substrate was used. Likewise, during the experiments, the anodic chamber of the reactor fed with synthetic wastewater (g L^−1^) contained glucose (1, 2, 5, & 10), K_2_HPO_4_ (1.4), KH_2_PO_4_ (0.25), NH_4_Cl (0.31), MgSO_4_ (0.1), KCl (0.13), NaCl (0.1), CaCl_2_ (0.1), and the mineral solution (0.1 mL).^55^

According to the results of previous studies,^63^ the anodic solution pH was regulated to 7 by adding phosphate buffer for improving microorganism activities in the seed sludge. Furthermore, before the operation of each run and adding inoculums, the anodic chamber was purged by N_2_ gas to make an anaerobic environment.^76^ The stable catholyte of the BEF reactor was prepared by adding 0.1 M Na_2_SO_4_ as supporting electrolyte and 10 mg L^−1^ TC into deionized water and continuously aerated to provide the dissolved oxygen required as an electron acceptor for *in situ* generation of H_2_O_2_ in the cathode chamber.^60^ Also, the pH of the catholyte was adjusted to the required values with 0.1 M diluted H_2_SO_4_ and NaOH solutions.^16^

All of the experiments were run in batch mode. In the first stage, the degradation efficiency of tetracycline under open-circuit conditions, close-circuit conditions, and short-circuit conditions in the cathode chamber of the BEF system were assessed. In further stages, the influence of different important operational parameters, including glucose as substrate (at different concentrations) in anolyte, solution pH (3, 5, and 7), FeSO_4_ concentrations (2, 5, and 10 mg L^−1^) in catholyte at 24 h reaction time was investigated to evaluate the efficiency of the BEF system in degradation of TC in an aqueous solution and energy generation. All experiments were carried out independently in duplicates at atmospheric pressure and controlled temperature of 30 ± 1 °C, and data were shown in terms of mean ± standard deviation (SD).

### 2.3. Output power measurement and calculation

The voltage output of the BEF system was automatically recorded using a digital data logger (Danesh Gostar Hamgam Ba Sanat Company, Iran) at 15 min intervals for 24 h and the personal computer connected to the system. When the maximum steady voltage was achieved, polarization and power density curves were determined by varying the different external resistances from 0.1 to 100 kΩ.^58,73^ Then, the power and current densities were normalized based on the employed surface area of the anodic electrode.^77^ The requirements were provided for the online observation of the polarization and power density curves, which reported the variation of voltage and power density according to the current density.

The current density (mA m^−2^) and power density (mW m^−2^) parameters were computed according to eqn (4) and (5), respectively:4
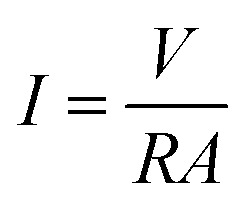
5
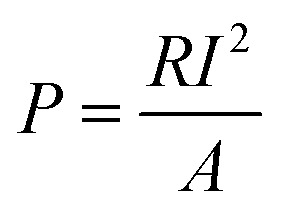
where *A* (15 cm^2^) is the anodic surface area, and *V* represents the cell voltage. Additionally, *R* and *I* indicate the external resistance and generated current, respectively. The current of the BEF system was calculated by dividing the achieved voltage by the specific resistance. Moreover, the obtained power by the BEF reactor was determined by multiplying the current and voltage.^55,63^

### 2.4. Chemical analytical methods

All samples were taken from the anode and cathode chambers of the reactor and were filtered through 0.45 μm filters before analysis. The pH of the solutions was measured using a digital pH meter (Philips PW 9422).^78^ The H_2_O_2_ concentration was analyzed using a UV-vis spectrophotometer (HACH DR5000) at 410 nm wavelength, with titanium(iv) oxysulfate as a coloured indicator (DIN 38402H15 method).^79^ The concentrations of residual total ion species of Fe^2+^ and Fe^3+^ were achieved using the 1,10-phenanthroline method at the end of operation time.^57^

The TC concentration was monitored by reversed-phase high-performance liquid chromatography (Agilent 1260 Infinity HPLC) equipped with a UV-VIS detector and C18 chromatographic column (4.6 mm × 100 mm, 3.5 μm) at a detected wavelength of 359 nm. The mobile phase involved an acetonitrile/oxalic acid solution (25 : 75% v/v) with an injection constant flow rate of 1 mL min^−1^ at 25 °C. Also, the injected volume of the sample was 20 μL.^80^ The TC degradation efficiency was calculated as:6
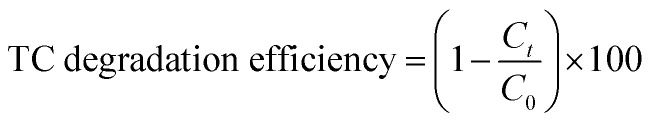
where *C*_0_ and *C*_*t*_ denote the TC concentrations before and after the process, respectively. The synthetic wastewater chemical oxygen demand (COD) was measured according to the colorimetric method with closed reflux at 600 nm (the Palintest system, photometer 5000, England).^81^

### 2.5. Characterization of the anode surface

The outer surface morphology of the anode was investigated using a field emission scanning electron microscope (FE-SEM, JEOL JSM-840A, Japan) at the voltage of 20 kV. For microscopic observations, the attached microorganisms on the surface of the carbon felt electrode was fixed overnight in a 2% (v/v) glutaraldehyde solution for 24 h at a temperature of 4 °C and pH of 7.5. Then, anode samples were fixed in 1.5% osmium tetroxide for 2 h and were dehydrated through water and alcohol solutions (30 to 70%, v/v 5 min each stage). Also, the samples were submerged for 5 min each in hexamethyldisilazane (50% (v/v) hexamethyldisilazane in 100% ethanol and 100% hexamethyldisilazane) and air-dried. In addition, after mounting the sample on a carbon film, it was coated with a gold layer using a Fullam sputter coater and finally scanned by SEM technique, and the size of an experiment specimen was 1 cm × 1 cm for SEM analysis.^55,58^

## 3. Results and discussion

### 3.1. *In situ* H_2_O_2_ concentration in the bio-electro-Fenton system

The H_2_O_2_ as the main Fenton reagent is a major component in the BEF system, and it is interesting to achieve H_2_O_2_ production.^60^ The H_2_O_2_ reagent is continuously produced by the electrochemical reaction of dissolved oxygen at the cathode, with electrons transferred from the microbial activity of the anode chamber in the BEF system.^82^ In addition, the variations in the values of H_2_O_2_ can show the levels of ˙OH for pollutant degradation by Fenton's reaction.^63^

**Fig. 2:**
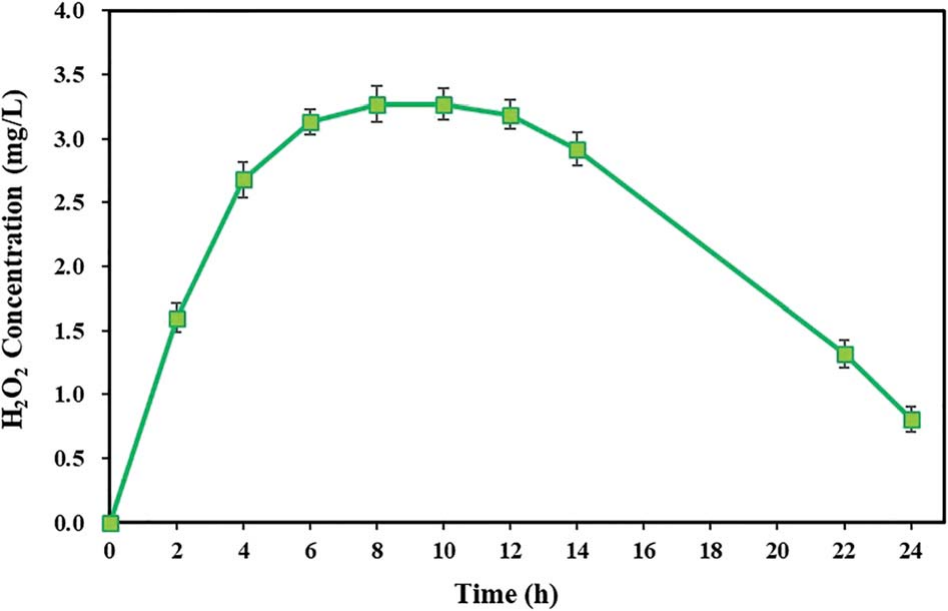
*In situ* H_2_O_2_ concentration in the cathode chamber of the BEF system.

According to the results of this study, TC degradation was observed with high degradation efficiency, indicating the presence of strong oxidants in the cathode chamber. To prove this, the cumulative concentration of detectable H_2_O_2_ in the cathode chamber of the BEF system was measured during a 24 h reaction time. As shown in Fig. 2, the H_2_O_2_ concentration increased over time and reached its maximum (3.27 ± 0.14 mg L^−1^) after 8 h. Then, it remained almost constant. In the next step, the H_2_O_2_ was consumed along with the TC degradation, and its concentration was reduced to 0.81 ± 0.1 mg L^−1^. Similar results were also achieved by Yong *et al.*^59^ They reported that by increasing the H_2_O_2_ concentration, its maximum cumulative concentration reached 135.96 μmol L^−1^ in 48 h, and then H_2_O_2_ concentration decreased after being consumed by Fenton's reactions. The study results of Wang *et al.*^60^ also showed that for both continuous and batch flow modes, the H_2_O_2_ generation had an increasing trend at first and reached the highest amount (2.1 mg L^−1^), but subsequently, it reduced.

The electrochemical generation of H_2_O_2_ in the BEF system generally consists of three main stages: the ascending production stage, the steady-state stage, and the descending stage. In the first stage, after starting the BEF system, the H_2_O_2_ reagent is exponentially generated in the cathodic chamber. Then, due to the equivalent generation and decomposition rates of H_2_O_2_ in the steady-state stage, it remains constant at its highest concentration. Finally, in the descending stage, the H_2_O_2_ concentration gradually reduces because of being consumed by Fenton's reaction, and consequently, the TC degradation rate decreases at the end of the process. Another reason for the decrease in H_2_O_2_ concentration is that the amount of electrons generated by the anodic degradation reduces *via* decreasing the substrate concentration during the process, resulting in a decline in the reaction rate of (eqn (1)).^59,83^

Generally, in the BEF system, *in situ* electrogenerated H_2_O_2_ as a strong oxidant is the most important factor because H_2_O_2_ is considered as a major source of ˙OH production in Fenton oxidation.^56^ Therefore, this alternative approach, due to its high efficiency in the generation of Fenton reagent (*e.g.*, H_2_O_2_) and the saving costs related to the storage and transport of chemicals, is advantageous in comparison to the other AOP processes, such as conventional Fenton process,^84^ UV/H_2_O_2_,^85^ photocatalysis,^45,46^*etc.*, in which H_2_O_2_ needs to be added.^15^ For example, Naushad *et al.*^86^ investigated the photo-degradation of methylene blue dye by applying different concentrations of H_2_O_2_. In another study, Yuan *et al.*^87^ pointed out that the addition of H_2_O_2_ is essential for more effective degradation of oxy-tetracycline, doxycycline, and ciprofloxacin in the UV/H_2_O_2_ process. Comparably, the promising BEF system has overcome some shortcomings of these methods.^69^

### 3.2. Bio-electro-Fenton system for TC degradation under different electrical circuit conditions

**Fig. 3:**
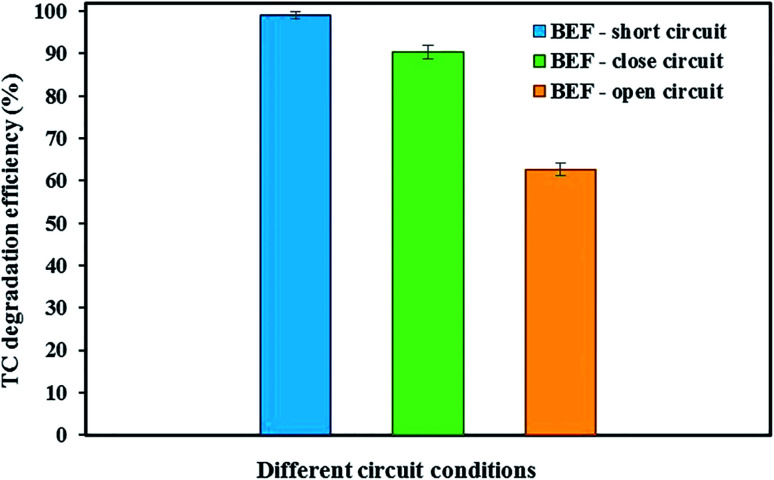
BEF system for TC degradation under different electrical circuit conditions.

Fig. 3 shows the degradation of TC in the cathode chamber of the BEF system under different conditions of open-circuit, close-circuit, and short-circuit with constant optimal conditions (anode chamber with a substrate concentration of 2000 mg L^−1^ and cathode chamber with tetracycline concentration of 10 mg L^−1^, cathode pH of 3, and FeSO_4_ concentration of 5 mg L^−1^) during 24 h reaction time.

When TC was used in the cathode chamber under open-circuit conditions (without external resistor load), only 62.68 ± 1.45% of the TC was removed. The reasons for low degradation of TC in open-circuit conditions were physical adsorption by the carbon felt cathodic electrode and also adsorption on the surface of the cathode chamber without Fenton's reactions causing TC degradation.^53,74^ Our results are in accordance with previous studies. Feng *et al.*^15^ showed that the orange II concentration in the BEF system decreased only by 3%, revealing that its adsorption on the electrode under the open circuit was insignificant. Wang *et al.*^60^ investigated the removal of emerging contaminants in the open circuit conditions of the MFC–Fenton system. They reported that the low removal of emerging contaminants is because of their adsorption by graphite rod electrodes and graphite granules without cathodic Fenton's reactions.

In the present study, compared with open-circuit conditions, the degradation efficiency of TC in the closed-circuit conditions of the BEF system with an external load of 1000 Ω increased by 27.62%, and the total degradation efficiency of TC was 90.30 ± 1.62%. Under such conditions, in addition to physical adsorption onto the carbon felt cathode electrode and the BEF reactor surface, H_2_O_2_ production in the cathode chamber was also responsible for TC degradation.^60^ In other words, Fenton's reactions based on the reaction of H_2_O_2_ with added Fe^2+^ generate more hydroxyl radicals, significantly enhancing the oxidation of the pollutant.^88^ Thus, the synergistic adsorption and catalytic degradation of antibiotics in the presence of highly reactive radicals can facilitate the degradation process simultaneously and accelerates the degradation rate.^47,60,61^ Similarly, Xu *et al.*^53^ achieved the removal of 81% of 17β-estradiol and 56% of 17α-ethynyl-estradiol in BEF system equipped with NCF (non-catalyzed carbon felt)/Fe@Fe_2_O_3_ cathode electrode in close circuit conditions and the adsorption onto the electrodes and reactor and also electro-Fenton reaction between the iron reagents (Fe^2+^) and H_2_O_2_ produced were the reasons for estrogen removal.

To achieve the maximum degradation efficiency, the degradation of TC was also studied under short-circuit conditions (at an external resistance of 0 Ω) of the BEF system. In such conditions, the degradation efficiency of TC improved compared to open- and closed-circuit conditions and reached 99.04 ± 0.91%. According to the previous studies, raising the cathodic current density increases the *in situ* production of H_2_O_2_ and subsequently promotes ˙OH formation from Fenton reaction to remove contaminants.^53,54,60^ Since in this experiment, the current density in short-circuit (695 mA m^−2^) was higher than the closed-circuit conditions (565 mA m^−2^), the concentration of detectable H_2_O_2_ in the steady-state of short-circuit conditions reached 3.27 ± 0.14 mg L^−1^ (refer Fig. 2), which was higher than the H_2_O_2_ amount measured in closed-circuit conditions (2.16 ± 0.14 mg L^−1^). As a result, this condition provided direct evidence for greater H_2_O_2_ generation at higher current density in the short-circuit, which was associated with the higher degradation efficiency of contaminants.^88^ Zhuang *et al.*^54^ studied the cathodic degradation of rhodamine B dye using a new bio-electro-Fenton process and showed that the rate of decolorization and mineralization of rhodamine B is dependent on the cathode current density. Also, a higher removal rate was gained with increasing current density under short-circuit compared to closed-circuit conditions, and about 95% of rhodamine B dye and 90% of TOC were removed in short-circuit conditions. Similar results were also observed in a study conducted by Xu *et al.*^88^ Moreover, the degradation of TC in the present study was comparable to the previous studies. For example, approximately 79.1% of TC was degraded within 7 days by an MFC system in a study by Wang *et al.*^18^ Also, Wu *et al.*^22^ achieved 56.7% TC degradation *via* photocatalytic degradation by TiO_2_ under visible (500 nm) light irradiation. Therefore, in the present study, maximum decomposition of TC was observed according to the results in the BEF system in comparison with other methods.

### 3.3. Effect of various initial substrate concentrations on the bio-electro-Fenton system performance

#### 3.3.1. Effect of various initial substrate concentrations on anodic COD removal efficiency

The organic carbon matter of wastewater in the anode chamber acts as an electron donor source in metabolic processes that cause substrate degradation and bioenergy production. Therefore, biodegradation of organic matter as a substrate takes place along with the transfer of electrons to the anode electrode under anaerobic conditions.^63,89^ At this stage, the performance of the BEF system in the COD removal of synthetic wastewater with variable substrate concentrations under optimal conditions (TC concentration of 10 mg L^−1^, cathodic pH of 3, and FeSO_4_ concentration of 5 mg L^−1^) with short circuit was investigated. In this regard, batch experiments were performed to optimize the conditions of the anode chamber to determine the maximum wastewater treatment.

As shown in Table 1, the removal efficiency of anodic COD varied between 83.32 ± 2.21% and 78.29 ± 2.68% during BEF system setup with different glucose concentrations (*e.g.*, 1000, 2000, 5000, and 10 000 mg L^−1^). The results showed that the COD removal efficiency increased with increasing the substrate concentration from 1000 to 2000 mg L^−1^ due to the favorable promotion of bioactivity of electrochemically active bacteria.^90^ The maximum anodic COD removal rate at the substrate concentration of 2000 mg L^−1^ was 85.71 ± 1.81%. Similarly, Rahmani *et al.*^55^ reported an increase in the COD removal efficiency from 53% to 78% by increasing the substrate concentration from 500 to 2000 mg L^−1^ in the MFC system.

**Table 1:** Effect of various initial substrate concentrations on the anodic COD removal efficiency and TC degradation efficiency in the cathode chamber

COD concentration (mg L^−1^)	Anodic COD removal efficiency (%)	TC degradation efficiency (%)
1000	83.32 ± 2.21	97.38 ± 1.24
2000	85.71 ± 1.81	99.04 ± 0.91
5000	80.51 ± 2.45	97.94 ± 1.31
10 000	78.29 ± 2.68	97.02 ± 1.42

However, with a further increase in substrate concentration, the removal efficiency of anodic COD decreased, and it reached 78.29 ± 2.68% at a substrate concentration of 10 000 mg L^−1^. In fact, with increasing concentration of anodic biodegradable organic carbons, the bio-oxidation reactions increased by electrogenic microorganisms.^55^ Furthermore, since the pH of 7 is favorable for the bioactivity of microorganisms in anaerobic environments, different reasons, such as the batch conditions, increasing the concentration of organic acids and intermediates produced from incomplete degradation of glucose, made the environmental conditions unsuitable for the growth and bioactivity of microbial population, so that the anodic pH reached 6.41 at 10 000 mg L^−1^ in the present experiment.^63,73,91^ In a study carried out by Gil *et al.*,^92^ the best microorganism activity was obtained at pH 7 in the MFC system, showing that microbial activity was slower at a lower pH. Hence, it can be stated that at very high concentrations of the substrate, COD removal efficiency slightly decreases. Similar studies have also revealed that there is a relationship between changes in substrate concentration and removal of anodic COD. For example, Zhang *et al.*^93^ declared that with increasing COD concentration, the residual COD concentration increases, indicating a low rate of COD removal at very high COD concentrations.

#### 3.3.2. Effect of various initial substrate concentrations on the TC degradation efficiency

The organic substrate is significant for the BEF because it influences not only the COD removal in the anodic chamber but the reactor performance, including the pollutant degradation efficiency.^74,94^ The degradation efficiency of TC in the cathode chamber under the steady optimal conditions was also examined with variable concentrations of the substrate. As shown in Table 1, in the BEF system, the TC degradation efficiency changed when the substrate concentration increased from 1000 to 10 000 mg L^−1^ for 24 h. Furthermore, by increasing the substrate concentration from 1000 to 2000 mg L^−1^, the degradation efficiency of TC varied from 97.38 ± 1.24% to 99.04 ± 0.91%. Accordingly, the value of 2000 mg L^−1^ was selected as the optimal concentration of the anodic substrate. The results also showed that with further increase in the concentration of anodic carbon source, the degradation efficiency had a descending trend, and the degradation efficiencies in the substrate concentrations of 5000 and 10 000 mg L^−1^ were determined to be 97.94 ± 1.31% and 97.02 ± 1.42%, respectively. Wang *et al.*^74^ obtained the same results by operating a microbial electro-Fenton system with variable concentrations of acetate as the anodic substrate. They reported that the removal efficiency of carbamazepine decreased by increasing the acetate concentration from 300 to 1000 mg L^−1^.

The best performance of the BEF system in substrate concentration of 2000 mg L^−1^ could be due to the decomposition of the appropriate concentration of H_2_O_2_ in the presence of ferrous ions for ˙OH generation, which, in turn, causes high degradation of TC.^16^ In addition, since the H_2_O_2_ generation was most probably attributable to the electricity generation, the electricity generation from the substrate utilization by electrochemically active bacteria in the anode increased the Fenton's reaction on the cathode in the BEF system, resulting in the reaction between TC and ˙OH.^74,83^ On the other hand, the decrease in degradation efficiency with increasing substrate concentration from 2000 to 10 000 mg L^−1^ may also result from the fact that with further increasing substrate concentrations, the current density of the system as generated electricity also increases; as a result, overproduction of H_2_O_2_ happens in the cathode chamber. So, the degradation efficiency at very high concentrations of H_2_O_2_ can reduce due to the scavenging effect of H_2_O_2_ (eqn (7) and (8)). High concentrations of H_2_O_2_ generate hydroperoxyl radicals (HO_2_˙) with a lower oxidation potential (*E*° = 1.65 V) than the hydroxyl radicals (*E*° = 2.80 V). These results are similar to the results of other studies that have shown a decrease in the efficient removal of organic pollutants at very high concentrations of H_2_O_2_ by producing hydroperoxyl radicals.^78,95^

7 H_2_O_2_ + ˙OH → HO_2_˙ + H_2_O

8 HO_2_˙ + ˙OH → H_2_O + O_2_

#### 3.3.3. Effect of various initial substrate concentrations on bioelectricity generation

Due to the direct transfer of electrons generated from the organic carbon matter degradation by bioactive microorganisms as biocatalysts attached to the anode, bioelectricity is generated using BEF technology. Therefore, the chemical energy produced from the anodic substrate oxidation is converted into bioelectrical energy.^18,96–98^

**Fig. 4:**
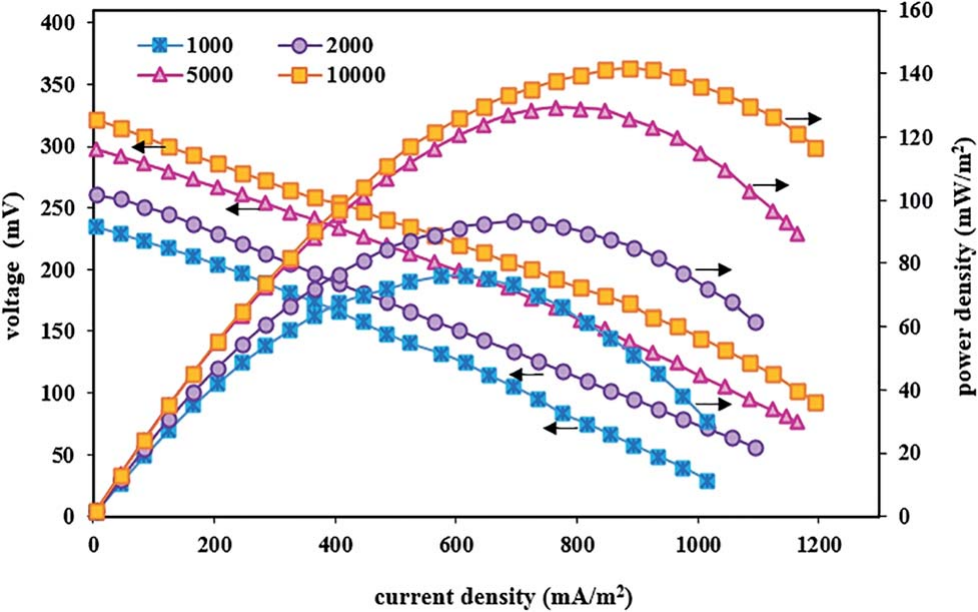
Polarization and power density curves of the BEF system in various substrate concentrations in the anode chamber.

In these fed-batch experiments, under optimal conditions of the cathode chamber and as well as steady-state conditions, polarization and power density curves were obtained after implementing the BEF system with variable substrate concentrations. Fig. 4 shows the effect of substrate concentration changes on voltage output, current density, and power density. The results revealed that the suitable concentration of the organic substrate was a major parameter influencing the performance of bioenergy production in the BEF system and the energy production showed a high dependence on the anodic substrate concentration.^77^ As can be seen in Fig. 4, increasing the substrate concentration has a positive effect on the performance of the BEF system in bioelectricity production, and the observed difference in the power, current, and voltage output is due to the difference in the substrate concentration. This result is consistent with the study of Rahmani *et al.*,^73^ who reported that the increase in substrate concentration in the anodic chamber of the MFC reactor has a positive effect on the voltage and power production due to the increased biological activities.

The system implemented with a high amount of substrate especially showed relatively good energy production, so that the maximum voltage, power density, and current density at the substrate concentration of 10 000 mg L^−1^ were 322 mV, 141.60 mW m^−2^, and 1195 mA m^−2^, respectively. Contrarily, in the substrate concentration of 1000 mg L^−1^, the maximum values of voltage, power density, and current density were 235 mV, 76.26 mW m^−2^, and 1015 mA m^−2^, respectively. In other words, the power density value increased with increasing substrate concentration while it declined at lower or higher current densities, and its maximum value produced higher current densities by increasing the substrate concentration. According to the obtained results, the significant relationship between all concentrations of the substrate with amounts of power and voltage output could be attributed to the electrochemical activity of electrogenic microorganisms attached to the anode, so that microbial activities increase with increasing substrate concentration, and the biochemical reaction of exoelectrogenic bacteria is completely conducted in the decomposition of organic substrate to generate and transfer electrons. This, in turn, increases the energy production by the system.^55,99–101^ Moreover, results of the enhancement of bioelectricity generation in the present study showed that microorganisms have adapted to the anodic solution conditions at high substrate concentrations and adding more carbon sources as electron donor has increased metabolism and growth of the bacterial community and has also affected the maximum power and voltage output with efficient electron transfer.^63,90^ Therefore, energy recovery from the organic substrate was achieved by the action of anodic microorganisms.^56^ Similar results with different substrate concentrations have been reported in previous studies.^92,93^ The results of a study by Wen *et al.*^101^ showed that increasing the substrate concentration (from the wastewater COD of 614 to 2062 mg L^−1^) had a significant positive effect on the electrochemical performance and electricity generation of the MFC system at higher current densities. They also reported that higher substrate concentration with produced COD of 2062 mg L^−1^ created suitable conditions for microbial growth in the anodic solution and increased the power density up to 42.6 W m^−3^. In another similar study, Gonzalez del Campo *et al.*^90^ reported that increasing the wastewater COD from 100 to 3000 mg L^−1^ enhanced the microorganism activities and as a result, the MFC system's current gradually increased. Therefore, the ability of energy production without the use of external energy supply is the most positive and significant advantage of the BEF process compared to other AOP processes with applied external energy, such as photo-Fenton,^102^ photo-electro-Fenton,^103^ ultrasound-Fenton,^104,105^ UV/H_2_O_2_,^106,107^ and electro-Fenton,^17,108^ which has attracted more and more attention in recent years.

### 3.4. Effect of initial catholyte pH on the TC degradation and bioelectricity generation

In the BEF system, pH is an important parameter affecting the Fenton oxidation efficiency. Accordingly, in this stage, the effect of different catholyte pH (3, 5, and 7) on the TC degradation as well as bioelectricity generation under optimal conditions of the TC concentration of 10 mg L^−1^, FeSO_4_ concentration of 5 mg L^−1^, and substrate concentration of 2000 mg L^−1^ (with the short-circuit) was investigated during 24 h.

**Fig. 5:**
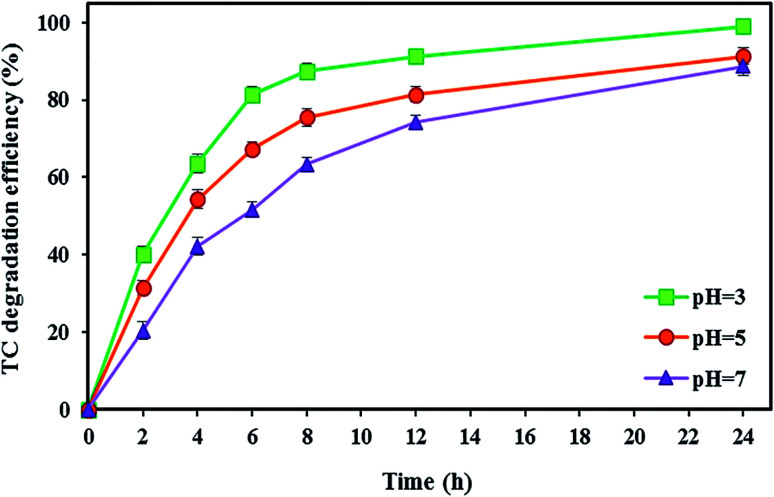
The effect of various catholyte pH values on the TC degradation in the BEF system.

Generally, Fenton's reaction is performed with maximum catalytic activity at an acidic pH of 3.^109^ As shown in Fig. 5, the maximum degradation efficiency of TC was 99.04 ± 0.91% at pH 3 after 24 h reaction time. Because Fenton's reaction was significantly affected by pH values, pH = 3 was considered an optimal value in the BEF system. This is because the maximum amount of available Fe^2+^ can be present in acidic media.^69^ Also, hydrogen peroxide is stable under acidic solution and *in situ* generated ˙OH as a reactive oxidant resulting from the degradation of stable H_2_O_2_ by Fenton's reaction according to eqn (2) increases the tetracycline oxidation efficiency.^110–112^ Moreover, ˙OH production may be increased by the catalytic action of the Fe^2+^/Fe^3+^ pair.^69^ This obtained result is similar to the findings of other reports. For instance, Zhang *et al.*^17^ investigated the degradation of tetracycline by the EF process and observed the maximum degradation of 84.6% at pH 3. Similarly, Hassan *et al.*^95^ reported that the microbial electro-Fenton system reached its complete degradation of sulfaquinoxaline, tylosin, and tetracycline at pH 3 in 24 h.

On the other hand, the performance of the BEF system decreased at high pH values, so that by increasing the pH value from 3 to 5, the TC degradation efficiency decreased by 7.76%, and with a further increase of pH from 5 to 7, the degradation rate reached 88.67 ± 2.32%. The decrease in efficiency at pH higher than 3 could be attributed to the conversion of Fe^2+^ to Fe^3+^ species, leading to the precipitation of Fe^3+^ as ferric hydroxide (Fe(OH)_3_) (eqn (9)), which can lead to a decrease in the amount of Fe^2+^ and catalyst deactivation and then the quantity of ˙OH decreases in the catholyte.^82^ Also, H_2_O_2_ is unstable at alkaline conditions and decomposes to H_2_O and O_2_ (eqn (10)).^50^ Moreover, there may not be enough protons to participate in the H_2_O_2_ formation at higher pH, or the catholyte acidity may not be adequate to prohibit the ferric hydroxide precipitation.^69^

9 Fe^3+^ + 3OH^−^ → Fe(OH)_3_

10 2H_2_O_2_ → O_2_ + 2H_2_O

This result was consistent with that reported previously. For example, Xu *et al.*^113^ reported that the maximum removal efficiency of rhodamine B at pH 3 reached 95% and significantly decreased with increasing pH in a BEF system. As a result, pH plays an important role in the TC degradation in BEF systems.

In the present study, pH values lower than 3 were not tested because the diffusion of more protons from the cathode compartment to the anode increases in an overly acidic environment that may decrease or even stop the bioactivity of anodic microorganisms. Then, these conditions reduce the production of bio-electricity. Thus, the cathodic Fenton's reaction terminates because of the cut-off of the electron input.^57,66^ Moreover, at lower pH, H_2_O_2_ cannot be decomposed to ˙OH *via* Fe^2+^. In this case, H_2_O_2_, by capturing one proton, converts to stable oxonium ion (eqn (11)) that is electrophilic and slows down the reaction rate between H_2_O_2_ and Fe^2+^.^78,114^

11 H_2_O_2_ + H^+^ → H_3_O_2_^+^

**Fig. 6:**
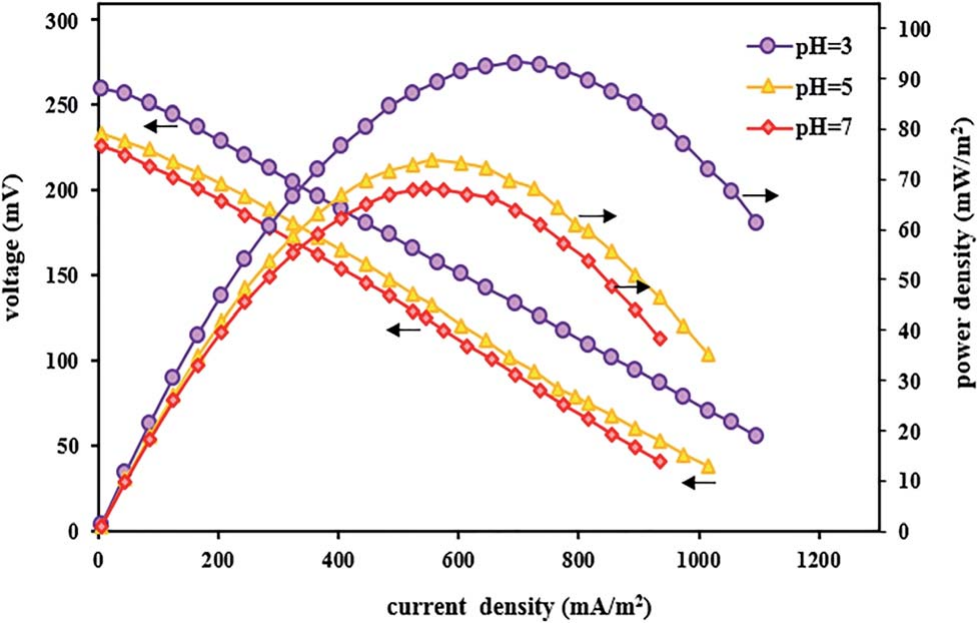
Polarization and power density curves of BEF systems with various catholyte pH values.

In addition to affecting the TC degradation efficiency, pH can also influence power output and voltage. By evaluating the polarization and power density curves at the optimal acidic pH of 3, the maximum power density and the maximum voltage were 93.13 mW m^−2^ and 260 mV, respectively (Fig. 6). The reasons for the high system performance were that the system takes advantage of the pH difference between the cathode and anode chambers and the fact that the over potential of the cathode chamber reduces under acidic conditions.^115^ Moreover, it was observed that by increasing the pH from 3 to 5 and 7, the power density and voltage output decreased, and the maximum power density and the maximum voltage at pH 7 were 68.12 mW m^−2^ and 226 mV, respectively. The same results were also reported by Luo *et al.*,^83^ where the power output increased with decreasing pH, and the maximum power was obtained at low pH values.

### 3.5. Effect of catholyte iron concentration on the TC degradation and bioelectricity generation

The iron catalyst concentration has a significant effect on Fenton's reaction efficiency. Adding FeSO_4_ as Fe^2+^ source to catholyte, which on combining with electrogenerated H_2_O_2_ can produce free ˙OH according to Fenton chemistry (eqn (2)).^69^ Moreover, reaction (eqn (2)) is reproduced by the electrochemical reduction of Fe^3+^ to Fe^2+^ through (eqn (12)) and continuous regeneration of Fe^2+^ ions in the cathode chamber. Also, some Fe^3+^ ions generated as a result of cathodic Fenton's reaction (eqn (2)) may be reduced to Fe^2+^ ions through eqn (13). Accordingly, Fe^2+^ ions can be continuously regenerated by Fe^3+^/Fe^2+^ redox cycle reproduction.^50,78^

12 Fe^3+^ + e^−^ → Fe^2+^

13 Fe^3+^ + H_2_O_2_ → Fe^2+^ + HO_2_˙

In this experiment, the effect of FeSO_4_ concentrations (2, 5, and 10 mg L^−1^) in the catholyte was evaluated on the degradation of TC antibiotic and electricity generation at initial TC concentration of 10 mg L^−1^, pH 3, and substrate concentration of 2000 mg L^−1^ (under short circuit conditions) during 24 h reaction time.

**Fig. 7:**
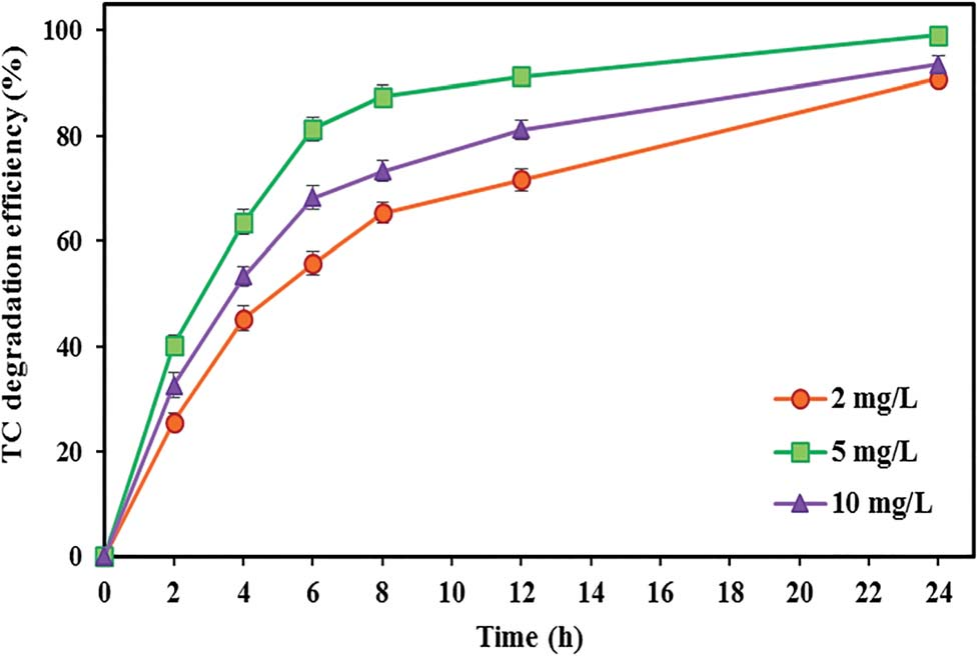
The effect of catholyte iron catalyst concentration on the TC degradation efficiency in the BEF system.

As shown in Fig. 7, increasing the FeSO_4_ concentration up to a certain optimal concentration increases the TC degradation efficiency. The degradation rate of TC increased by around 8.25% with increasing FeSO_4_ concentration from 2 to 5 mg L^−1^, and the maximum degradation efficiency of TC (99.04 ± 0.91%) was observed at the optimal FeSO_4_ concentration of 5 mg L^−1^ after 24 h. Zhang *et al.*^57^ also applied a similar amount of optimal FeSO_4_ concentration, *i.e.*, 5 mg L^−1^, for the iron catalyst. Ferrag-Siagh *et al.*^14^ also reported that the mineralization efficiency of TC in the EF process depended on the ferrous ion concentration, and increasing the Fe^2+^ concentration enhanced mineralization by about 10%. Maximum decomposition rate at an iron concentration of 5 mg L^−1^ might be attributed to the highest ˙OH production in the BEF system so that the amount of ˙OH produced could be increased by increasing the Fe^2+^ concentration according to the main Fenton's reaction (eqn (2)).^57,116^ Similarly, Fu *et al.*^67^ reported that the degradation efficiency of amaranth dye increased by changing the iron concentration from 0.1 to 1 mg L^−1^ due to the increase in ˙OH, and they determined the concentration 1 mg L^−1^ as the optimal Fe^2+^ concentration. Wang *et al.*^117^ revealed that COD removal from dyeing wastewater increased with added Fe^2+^ concentrations from 0.33 to 2 mM in the EF process.

In contrast, the degradation efficiency decreased to 93.46 ± 1.64%, with a further increase in the iron concentration from 5 to 10 mg L^−1^. The negative effect of increasing the catalyst concentration on the degradation rate may be attributed to the increase in the occurrence of adverse parasitic reactions between the ˙OH and the excess Fe^2+^ so that the excessive Fe^2+^ leads to ˙OH loss (eqn (14)), thereby decreasing the removal efficiency.^50,82^ Also, with overproduction of ˙OH due to excessive presence of Fe^2+^ in solution (eqn (2)), the reaction between ˙OH and H_2_O_2_ occurs (eqn (7)), and consequently the generated hydroperoxyl radicals have negative effects on the efficiency of Fenton's reactions.^67^ This was consistent with the study of Annabi *et al.*,^33^ who applied an EF system for degradation of enoxacin antibiotic and found that further increase in the Fe^2+^ concentration in the cathode resulted in a decrease of degradation rate due to the competitive reactions occurring between ˙OH and the excess Fe^2+^ ions.

14 Fe^2+^ + ˙OH → Fe^3+^ + OH^−^

**Fig. 8:**
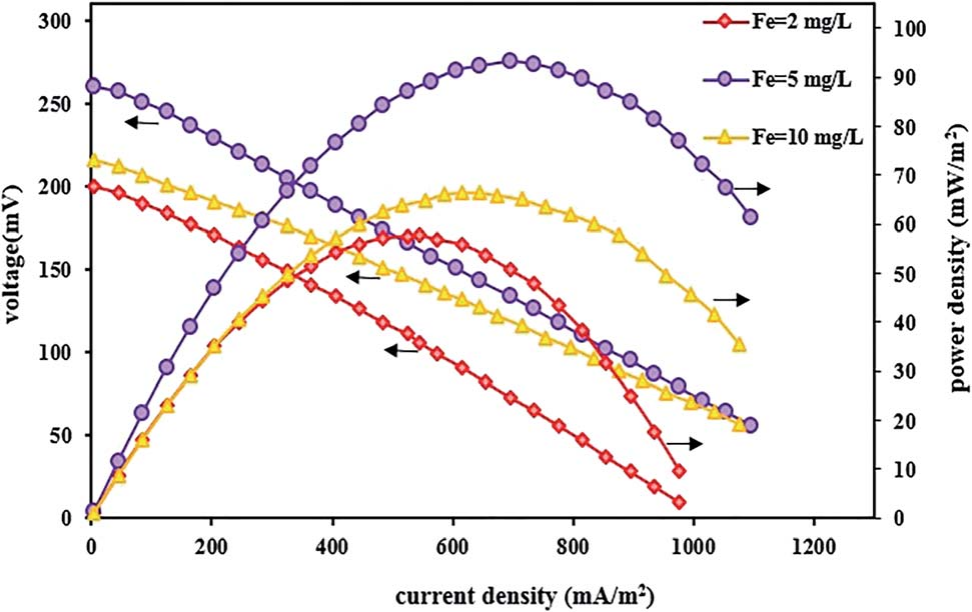
Polarization and power density curves of BEF systems with various catholyte iron catalyst concentrations.

In this section, the effect of changing the FeSO_4_ concentration was also investigated on the system performance for bioelectricity generation by setting up a BEF system with different FeSO_4_ concentrations and the changes in power and voltage output were reported by changing the iron concentration in the cathode solution. Fig. 8 shows the effect of various iron concentrations on the power and voltage output. As can be seen from the polarization and power density curves, the power and voltage output increase by raising the iron concentration from 2 to 5 mg L^−1^. The system with an iron concentration of 5 mg L^−1^ had better performance due to the increase in the concentration of iron ions so that the maximum voltage reached 260 mV and the maximum power density was 93.13 mW m^−2^ at a current density of 695 mA m^−2^. Then, with a further increase in iron concentration up to 10 mg L^−1^, the power density and voltage decreased to 66.44 mW m^−2^ and 216 mV, respectively. Therefore, concentration values higher than 5 mg L^−1^ had an adverse effect on system performance. A similar result was also reported by Wang *et al.*^70^ that the BEF system performed better with increasing iron concentration. As a result, the cell power and voltage output were increased. Also, in another study, Luo *et al.*^83^ found that the iron dosage influenced the maximum power density in the MFC–Fenton system, and increasing the FeVO_4_ dosage changed the maximum power in the range of 15.3–16.1 W m^−3^.

### 3.6. Microbial morphology of the anode

**Fig. 9:**
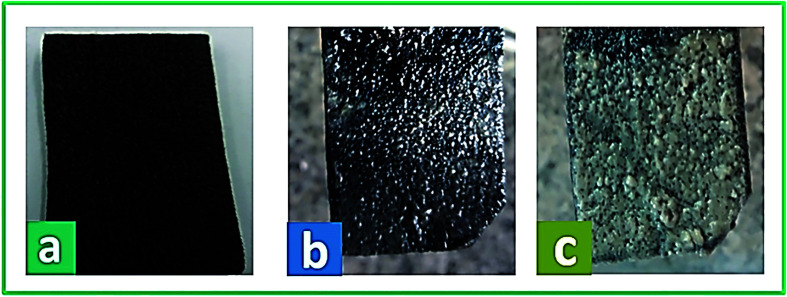
The real images of bare carbon felt (a) and biofilm formation on carbon felt electrode at the start (b) and end of the operation (c).

**Fig. 10:**
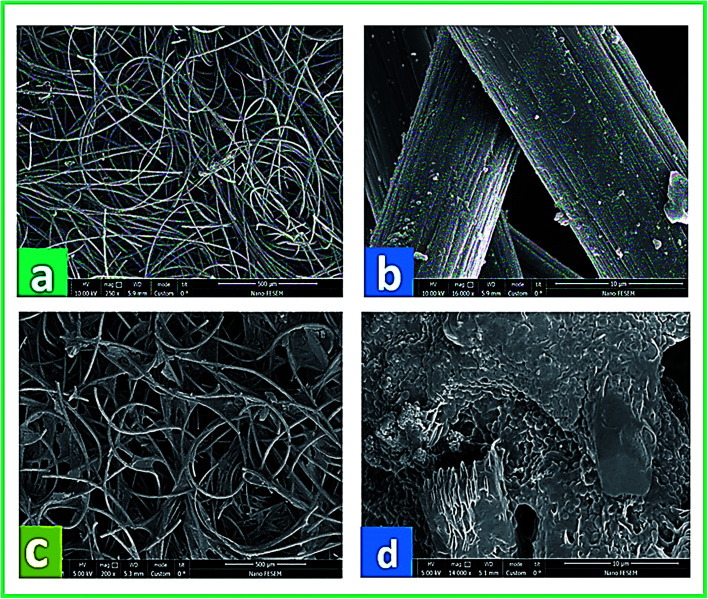
FE-SEM micrographs from the carbon felt surface before (a and b) and after (c and d) utilization in the anode chamber of the BEF system (large and close view).

The physical morphology of the anode and the growth of active microorganisms on the carbon felt surface were investigated by FE-SEM. In Fig. 9a, b, and c, the real images of the bare carbon felt electrode and the biofilm formation on the carbon felt electrode at the start and end of system operation are shown, respectively. Besides, Fig. 10a–d presents the SEM micrographs of the anode surface before and after usage in the BEF system (large and close view). According to Fig. 10a and b, the porous structure of the carbon felt electrode provides a high active surface area for bacterial biofilm formation. The SEM observations illustrated the complete coating of both sides of the carbon felt surface by the growth of microorganisms at the end of the process in a large and close view (Fig. 10c and d). According to the above analysis, the anodic COD removal, TC degradation, and electricity generation in the BEF system result from the suitable growth of anaerobic microbial communities on the electrode surface. Similarly, in a study by de Dios *et al.*,^118^ SEM images revealed microbial colonization on the graphite anode electrode of the benthonic MFC system integrated with an EF process. Also, the SEM analysis in a study by Yu *et al.*^119^ determined the coverage of the biofilm layer on the anode electrode in the MFC system.

According to the above analysis, the suitable growth of anaerobic bacterial communities on the electrode surface can result in appropriate performance of the BEF system. Therefore, a mixture of various types of electrochemically active bacteria was needed that was prepared with anaerobic sludge.^55^ The results of previous studies showed that Shewanella, Geobacter, Arcobacter, Comamonas, Dechloromonas, Sphingobacterium, Desulfobulbaceae, Firmicutes, and Clostridium were well-known and dominant electrochemically active bacterial species in mixed culture biofilm that attach to the anodic electrode and form a dense biofilm layer.^50,56,120^

## 4. Conclusions

The BEF system as a promising technology represents a new approach without the requirement of the external electricity supply for anodic COD removal and cathodic TC degradation with bioelectricity generation at the same time. The degradation efficiency for TC by BEF system with short circuit was higher than that of BEF with open and closed circuit. 99.04 ± 0.91% degradation of TC was achieved within 24 h under the conditions of short circuit, initial pH of 3, FeSO_4_ concentration of 5 mg L^−1^ in cathode chamber and substrate concentration of 2000 mg L^−1^ in the anode chamber. The high degradation of TC antibiotic in the cathode chamber was related to ˙OH generated from Fenton's reaction and also the adsorption onto the reactor and carbon felt electrodes.

The substrate concentration affected the BEF performance in anodic COD removal, TC degradation, and also bioelectricity generation. In the BEF system, a very high concentration of substrate (*e.g.*, 2000 to 10 000 mg L^−1^) resulted in lower efficiency of COD removal and TC degradation, while the highest bioenergy production was obtained. The BEF system produced the maximum current and power densities of 1195 mA m^−2^ and 141.60 mW m^−2^ according to the polarization curves, respectively. This work confirmed that the BEF system is an energy-saving and environmentally friendly method for the efficient treatment of antibiotic wastewater.

## Conflicts of interest

There are no conflicts to declare.

## Supplementary Material
